# CD39^+^ regulatory T cells accumulate in colon adenocarcinomas and display markers of increased suppressive function

**DOI:** 10.18632/oncotarget.26435

**Published:** 2018-12-11

**Authors:** Filip Ahlmanner, Patrik Sundström, Paulina Akeus, Jenny Eklöf, Lars Börjesson, Bengt Gustavsson, Elinor Bexe Lindskog, Sukanya Raghavan, Marianne Quiding-Järbrink

**Affiliations:** ^1^ Department of Microbiology and Immunology, The Sahlgrenska Academy at University of Gothenburg, Göteborg, Sweden; ^2^ Department of Surgery, The Sahlgrenska Academy at University of Gothenburg, Göteborg, Sweden

**Keywords:** CD39, regulatory T cells, colon cancer, adenosine, immune checkpoint molecules

## Abstract

Increasing knowledge of the function and regulation of tumor-infiltrating lymphocytes has led to new insights in cancer immunotherapy. Regulatory T cells (Treg) accumulate in colon tumors, and we recently showed that CD39^+^ Treg from cancer patients inhibit transendothelial migration of conventional T cells. CD39 mediates the hydrolysis of ATP to immunosuppressive adenosine and adds to the immunosuppressive effects of Treg. Here, we further investigated the regulatory features of intratumoral CD39^+^ Treg in colon cancer. Using flow cytometry analyses of cells from 46 colon cancer patients, we confirm the accumulation of CD39^+^ Treg in the tumor tissue compared to unaffected colon tissue, and also show that tumor-infiltrating Treg express more CD39 and Foxp3 on a per cell basis. Furthermore, CD39^+^ Treg in tumors express markers indicating increased turnover and suppressive ability. In particular, tumor-infiltrating CD39^+^ Treg have high expression of surface molecules related to immunosuppression, such as ICOS, PD-L1 and CTLA-4. Functional suppression assays also indicate potent suppressive capacity of CD39^+^ Treg on proliferation and IFN-γ secretion by conventional T cells. In conclusion, our results identify tumor-infiltrating CD39^+^ Treg as a numerous and potentially important immunosuppressive subset, and suggest that immunotherapy aimed at reducing the activity of CD39^+^ Treg may be particularly useful in the setting of colon cancer.

## INTRODUCTION

During recent years new insights in the process of tumor initiation and progression have emphasized the importance of the immune system and especially tumor-infiltrating immune cells in reducing tumor growth [1, 2]. The composition of tumor infiltrating immune cells changes with tumor growth and the nature of the infiltrate is closely associated with patient survival [3]. The tumor immune infiltrate may consist of both anti- and pro-tumorigenic immune cell subsets, and massive efforts have been invested in characterizing the tumor immune response, not least against colorectal cancer (CRC). Importantly, such studies show that integrative analyses of the immune cell infiltrate is in fact a better predictor of patient survival than tumor stage and microsatellite instability (MSI) [4]. In CRC, most of the tumor-infiltrating T cell subsets are associated with improved patient survival, still the tumor immune infiltrate is a complex network where the different subsets will give distinct contributions [3]. In addition, the genetic profile of the tumor also impacts the infiltration and function of the different immune cell subsets [5, 6]. Yet another interesting trait for CRC is the effect of the microbiota on the adaptive immune system and the increased accumulation of T helper 17 cells (Th17 cells) in CRC compared to other cancer forms [7]. This is particularly important, as a dominant Th17 response has been shown to be associated with poor survival in CRC, while T helper 1 cells (Th1 cells) and cytotoxic T lymphocytes (CTLs) are associated with improved survival [3, 8].

It is now well established that regulatory T cells (Treg) accumulate in most solid tumors, among them colorectal adenocarcinomas [3, 9–11], and that an increased Treg number in the tumor usually correlates with increased mortality [12]. Treg can exert immunosuppressive function through several mechanisms [13] and specific immunosuppressive mechanisms of intratumoral Treg in CRC towards effector T cells are not completely explored. So far PD-L1-, IL-10- and TGF-β-signaling have been associated with Treg immunosuppressive function in CRC [14, 15]. The effect of Treg on patient outcome in CRC has until recently been contradictory [8, 10, 16, 17] but a recent publication by Saito *et al.* has shed light on this controversy, indicating two different CD4^+^Foxp3^+^ T cell populations of which only one comprise truly suppressive Treg. The distribution of these populations vary between tumors and correlate to patient outcome [18].

To fully understand the interplay between tumor infiltrating Treg and other T cell subsets in CRC, further characterization of different tumor infiltrating Treg subsets is of essence. To this end, we and others recently showed increased frequencies of Treg expressing CD39 in colon or colorectal adenocarcinomas [9, 15, 19] and similar observations have also been made in head and neck squamous cell carcinoma and autoimmune diseases [20–22]. CD39 is an ectoenzyme that mediates hydrolysis of ATP to immunosuppressive adenosine. Sequential hydrolysis of ATP to adenosine is catalyzed by the two ectoenzymes CD39 and CD73, and when pro-inflammatory ATP is released into the extracellular space upon inflammatory stress or tissue damage, CD39 plays an important role in controlling the extracellular levels of ATP [23]. CD39 is thus a key molecule in the regulation of purinergic signaling and balances pro- and anti-inflammatory signals. Among lymphocytes, CD39 is predominantly expressed by Treg, and adenosine is an important Treg effector molecule which acts on conventional T cells and antigen-presenting cells, without decreasing the suppressive ability of the Treg itself [24, 25]. Apart from this direct effect of adenosine on effector T cells, our previous study also showed that CD39^+^ Treg from colon cancer patients have the ability to suppress transendothelial migration of conventional T cells, affecting anti-tumor immunity in yet another way [9]. In the current study, we examined CD39^+^ Treg isolated from tumors and unaffected mucosa from colon cancer patients to elucidate their contribution to immunoregulation and tumor progression. We now show that CD39^+^ Treg are enriched in colon tumors and have a phenotype indicating increased immunoregulatory functions and higher proliferation. Thus, we propose that tumor-infiltrating CD39^+^ Treg have a critical impact on anti-tumor immunity.

## RESULTS

### Treg accumulate in tumor tissue and have increased CD39 expression

**Figure 1:**
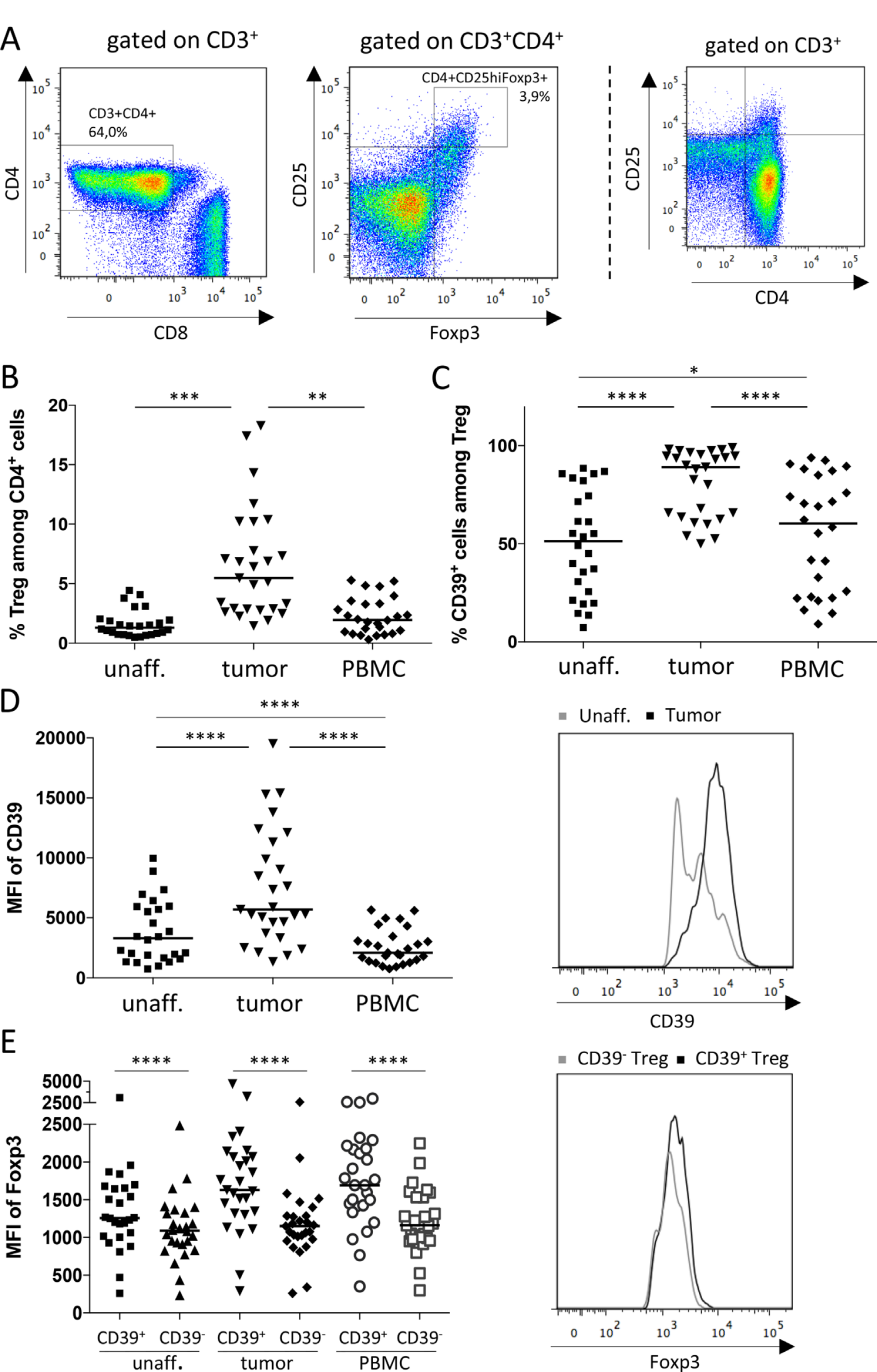
Frequencies of Treg and CD39^+^ Treg in colon adenocarcinoma patients Single-cell suspensions were isolated from unaffected colon lamina propria, tumors and PBMC. Treg and conventional T cells were identified by flow cytometry using an isotype control to determine cut-off for Foxp3 expression. The gating strategy is shown in (**A**), depicting a cell suspension from tumor tissue, and a FACS-plot to aid for gate placement for CD25^hi^ expression is shown to the far right. Frequencies of Treg among CD4^+^ cells (**B**), frequencies of CD39^+^ Treg (**C**), mean fluorescence intensity (MFI) of CD39 expression by CD39^+^ Treg (**D**), and MFI of Foxp3 expression by CD39^+^ and CD39^−^ Treg (**E**), was determined by flow cytometry in single cell suspensions from tumors, unaffected colon tissue and peripheral blood. Histograms display fluorescence for selected markers by individual cells from unaffected colon tissue and tumor (D), and from tumor (E). Symbols represent individual values and horizontal lines the median (*n* = 28). ^*^p < 0.05, ^**^p < 0.01, ^***^p < 0.001, ^****^p < 0.0001.

Previous studies have shown an accumulation of Treg in colorectal tumors [10, 11], and recently it was also demonstrated that many of these cells express the ectoenzyme CD39 [9, 15, 19]. As our recent findings indicate CD39^+^ Treg from cancer patients as a key subset in the inhibition of effector T cell transendothelial migration [9], we initiated studies to further define the role of this subset in the tumor microenvironment. Single cell suspensions from blood, colon tumors and unaffected colon tissue from the same patient were analyzed by flow cytometry. Treg were identified as CD4^+^Foxp3^+^CD25^high^ T cells using conventional gating strategies [26] (Figure 1A). Also in this patient material, Treg were found to accumulate in colon tumors compared to unaffected tissue (Figure 1B). We could also confirm that the frequencies of CD39^+^ Treg were higher in the tumors than in unaffected tissue or peripheral blood (Figure 1C). Furthermore, not only is the ratio between the CD39^+^ and the CD39^−^ Treg higher in the tumor tissue compared to unaffected tissue, but the CD39^+^ Treg in the tumor also have a higher density of CD39 on the cell surface than CD39^+^ Treg in unaffected tissue (Figure 1D). In addition, CD39^+^ Treg in the tumor have higher density of intracellular Foxp3 than CD39^−^ Treg (Figure 1E), indicating a higher level of differentiation and activity [18]. In this context, it was also interesting to characterize CD39^+^ and CD39^−^ Treg in relation to their expression of CD45RA and Foxp3. As shown by Miyara *et al.* [27], the CD4^+^ T cell fraction from peripheral blood can be divided into several functionally distinct subpopulations by their expression of CD45RA and Foxp3; among them CD45RA^+^Foxp3^lo^ resting Treg, CD45RA^−^Foxp3^hi^ activated Treg, and cytokine-secreting CD45RA^−^Foxp3^lo^ non-suppressive T cells. In line with the higher density of Foxp3 on the CD39^+^ Treg population in the tumor (Figure 1E), our data indicate that CD39^+^ Treg more frequently belong to the CD4^+^CD45RA^−^Foxp3^hi^ activated Treg population, compared to CD39^−^ Treg (Supplementary Figure 1).

### CCR6 and CCR10 are enriched on intratumoral CD39^+^ Treg

A selective recruitment of CD39^+^ Treg into the tumor could contribute to their increased accumulation, and we were interested in determining the process of recruitment of different Treg subsets into the tumors. We examined the expression of the chemokine receptors CCR4, CCR5, CCR6, CCR8, CCR9, CCR10 and CXCR3 by CD39^+^ and CD39^−^ Treg from the different tissue locations. We found that CCR6 and CCR10 were the only chemokine receptors that were more highly expressed by the CD39^+^ Treg in the tumor compared to the CD39^−^ Treg (Supplementary Figure 2). These chemokine receptors have been implicated in Treg homing to tumors [28–30], and in blood CD39^+^ Treg also expressed much more CCR10 than CD39^−^ Treg. Several of the chemokine receptors were expressed at similar levels in tumor and unaffected colon, but were more divergent in peripheral blood. The density of the selected chemokine receptors on each individual Treg in colon tissue and tumors was also similar between the subsets (data not shown). To conclude, except for CCR6 and CCR10, CD39^+^ and CD39^−^ Treg in tumors have a largely similar chemokine receptor expression, and chemokine receptor usage alone can probably not explain the preferred accumulation of CD39^+^ Treg in the tumor.

### CD39^+^ Treg show markers of increased activation

**Figure 2:**
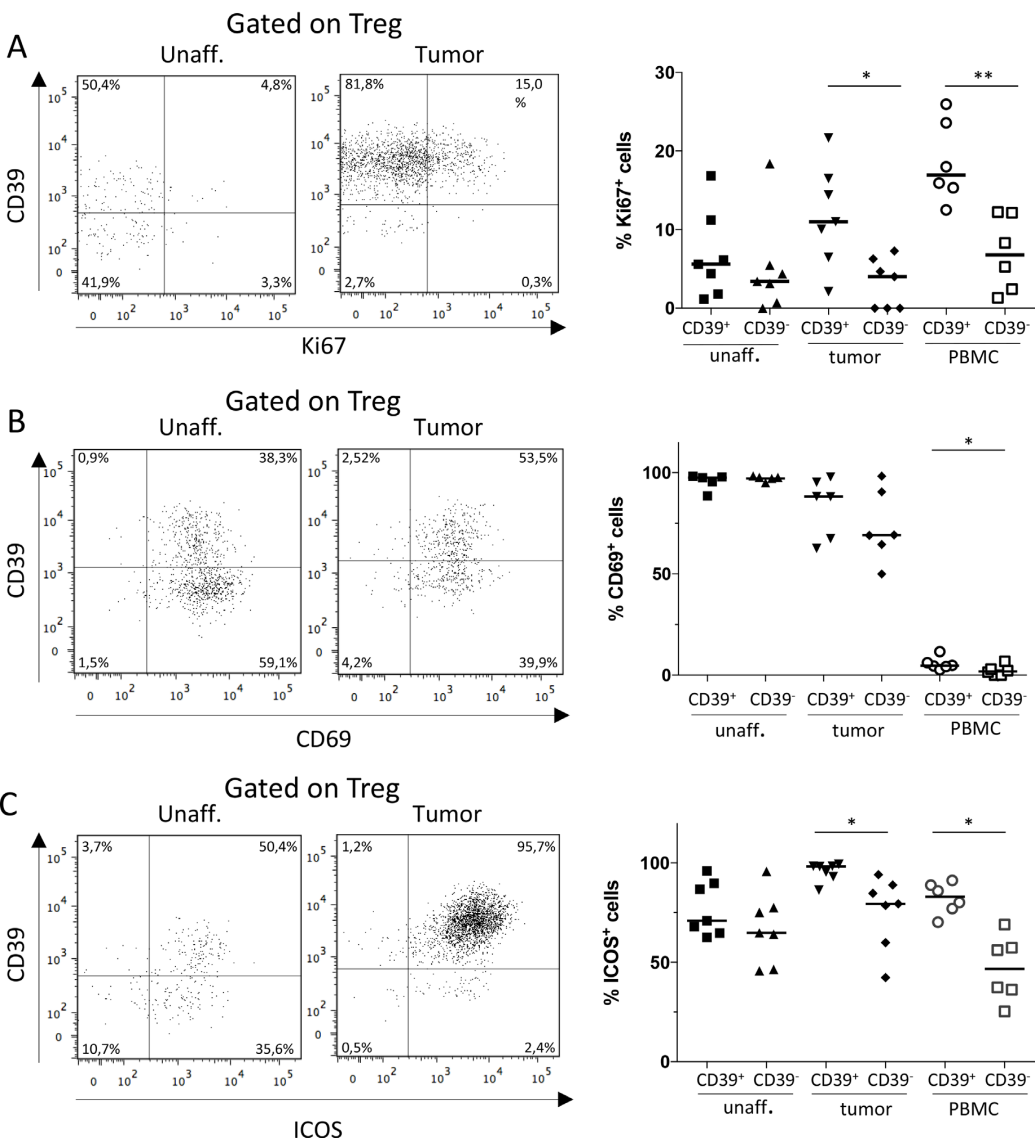
Expression of Ki67, CD69 and ICOS by CD39^+^ and CD39^−^ Treg Single-cell suspensions were isolated from unaffected colon lamina propria, tumor, and PBMCs. CD39+ and CD39- Treg were analyzed for their expression of Ki67 (**A**), CD69 (**B**) and ICOS (**C**), by flow cytometry using isotype controls to determine the cut-off for CD69 and ICOS expression. Representative FACS-plots gated on Treg from unaffected colon lamina propria and tumor are shown to the left, and a compilation of data to the right. Symbols represent individual values and horizontal lines the median (*n* = 7). ^*^p < 0.05, ^**^p < 0.01.

To further investigate the functions of CD39^+^ Treg in colon tumors and their activation stage, we started by examining the proliferation of CD39^+^ and CD39^−^ Treg populations by means of Ki67 expression. The frequencies of Ki67^+^ Treg ranged from 0 to 26%, with the highest expression in the CD39^+^ Treg population from peripheral blood. The CD39^+^ Treg population showed significantly higher proliferation in peripheral blood as well as in the tumor, compared to the CD39^−^ Treg population (Figure 2A). CD69 is also expressed on recently activated T cells and there was a trend that the CD39^+^ Treg in the tumor had higher expression of CD69. However, Treg expression of CD69 was near 100% in unaffected colon tissue and only slightly lower in the tumor (Figure 2B). CD69 is also a marker for tissue-resident memory T cells [31] and it is thus not likely that all CD69^+^ Treg in colonic tissues are recently activated. Instead the data may indicate that the tumor Treg population contain somewhat fewer tissue resident memory cells. We also examined the colon Treg populations for expression of inducible costimulatory molecule (ICOS, also known as CD278), a costimulatory surface molecule preferentially expressed on activated T cells and a marker for highly suppressive antigen-specific T cells [32, 33]. The CD39^+^ Treg population in the tumor, as well as in peripheral blood, showed significantly higher expression of ICOS compared to the CD39^−^ Treg population in the same tissue. In addition, the highest and most consistent ICOS expression was found on the tumor-infiltrating CD39^+^ Treg (Figure 2C).

Taken together, these data indicate that the CD39^+^ Treg subset in colon tumors is more dynamic compared to the CD39^−^ Treg subset, with a higher degree of proliferation and a phenotype indicating a higher suppressive ability on a population basis.

### CD39^+^ Treg express higher levels of immunosuppressive surface molecules than CD39^−^ Treg

**Figure 3:**
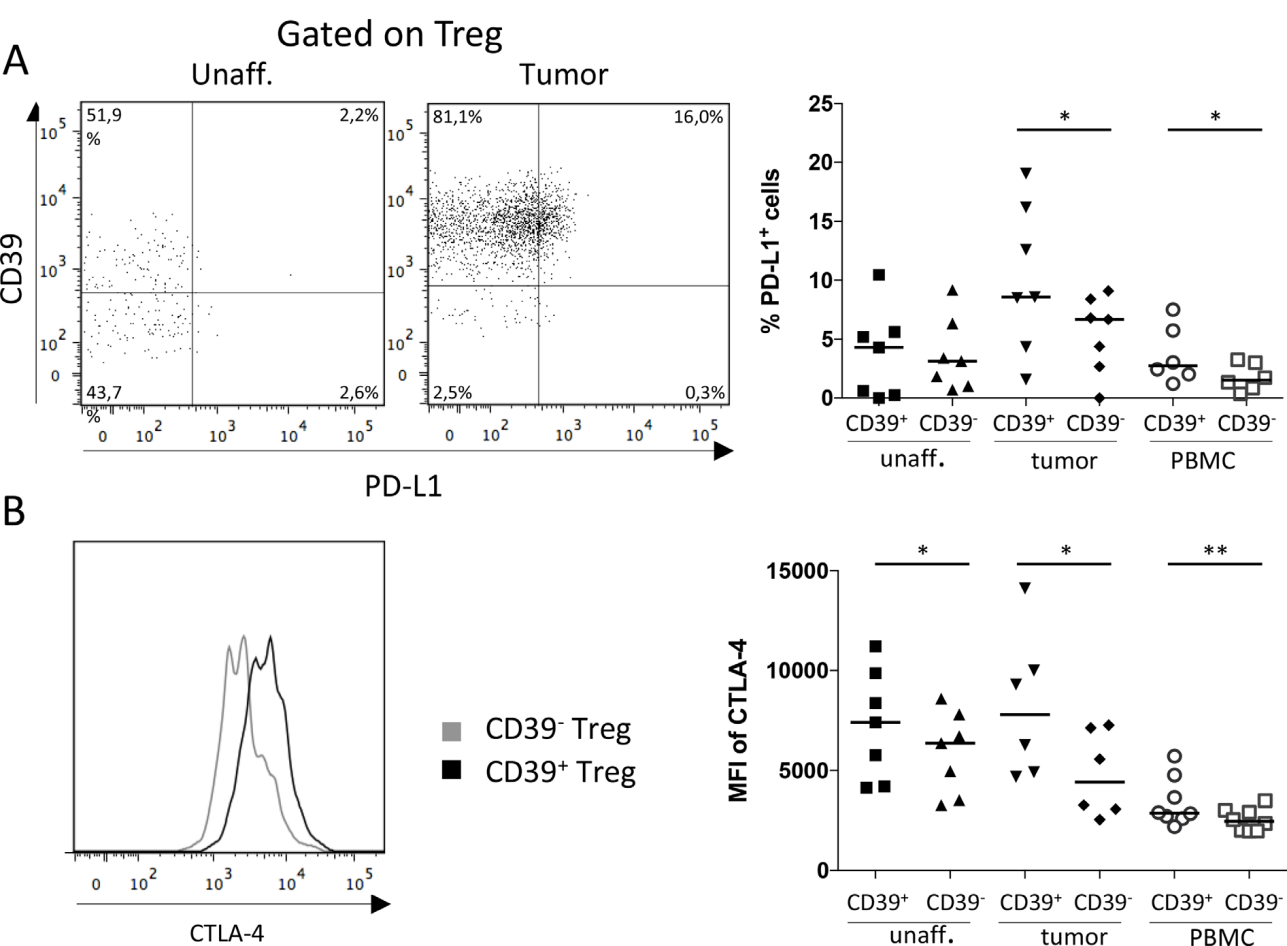
Expression of PD-L1 and CTLA-4 by CD39^+^ and CD39^−^ Treg Single-cell suspensions were isolated from unaffected colon lamina propria, tumor, and PBMCs. CD39^+^ and CD39^−^ Treg were analyzed for frequencies of PD-L1-expressing cells (*n* = 7; **A**) and MFI of CTLA-4 expression (*n* = 8; **B**), by flow cytometry using isotype controls to determine the cut-off for PD-L1 and CTLA-4 expression. Representative FACS-plots showing PD-L1 expression gated on Treg from unaffected colon lamina propria and tumor are shown to the left, and a compilation of data to the right (A). Mean fluorescence intensity (MFI) of CTLA-4 expression was analyzed on CD39^+^ and CD39^−^ Treg (B). A representative histogram showing CTLA-4 expression on CD39^+^ and CD39^−^ Treg from a tumor is shown to the left, and a compilation of data to the right. Symbols represent individual values and horizontal lines the median. ^*^p < 0.05, ^**^p < 0.01.

With the knowledge that activated CD39^+^ Treg accumulate in colon tumors, we wanted to examine if the expression of CD39 by Treg also correlate with the expression of other immunosuppressive effector molecules. We thus examined tumor-associated Treg for expression of the immunosuppressive surface molecule programmed death-ligand 1 (PD-L1, also known as CD274), which binds to its receptor, programmed death-1 (PD-1), on T cells and confers an inhibitory signal repressing T cell immunity [14]. We could demonstrate significantly higher expression of PD-L1 in the CD39^+^ Treg population compared to the corresponding CD39^−^ Treg population in the tumor tissue (Figure 3A). This was also true for peripheral blood. In contrast, no significant difference in PD-L1 expression was observed between the Treg populations in unaffected colon tissue. Comparing all three tissues, Treg from tumor tissue showed the highest expression of PD-L1. Since the tumor tissue is also enriched for CD39^+^ Treg, this further contributes to a higher number of total PD-L1-expressing Treg in the tumor compared to the other tissues.

In the same set of experiments, we also examined the expression of cytotoxic T-lymphocyte-associated protein 4 (CTLA-4, also known as CD152). CTLA-4 is a co-inhibitory receptor on conventional CD4^+^ and CD8^+^ T cells, conferring immunosuppression, whilst on Treg it is an effector molecule instead promoting further suppressive capacity [12]. The density of CTLA-4 was significantly higher on the CD39^+^ Treg compared to the CD39^−^ Treg in all three tissues (Figure 3B), and the difference in density between the subsets was largest in tumor-infiltrating Treg. Taken together, surface marker expression suggests that CD39^+^ Treg constitute a more immunosuppressive subset compared to CD39^−^ Treg, especially in the tumor microenvironment.

### CD39^+^ and CD39^−^ Treg display similar cytokine production

**Figure 4:**
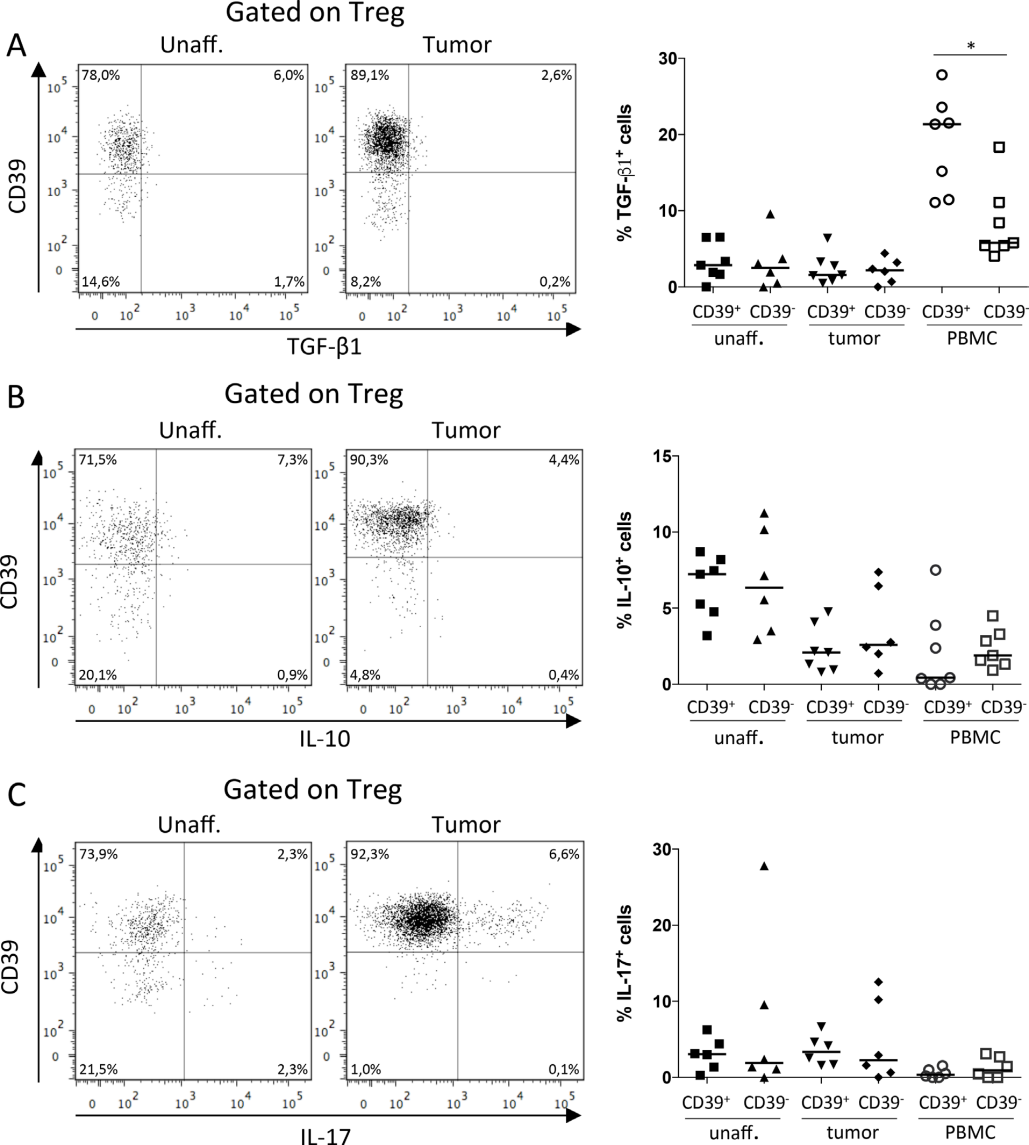
Production of TGF-β1, IL-10 and IL-17 by CD39^+^ and CD39^−^ Treg Single-cell suspensions were isolated from unaffected colon lamina propria, tumor, and PBMCs, and stimulated with PMA/Ionomycin. CD39^+^ and CD39^−^ Treg were analyzed for frequencies of TGF-β1 (*n* = 7; **A**), IL-10 (*n* = 7; **B**), and IL-17 (*n* = 6; **C**) producing cells by flow cytometry using unstimulated cells as reference to determine cytokine-specific staining. Representative FACS-plots showing data from unaffected colon lamina propria and tumor are shown to the left, and a compilation of data to the right. Symbols represent individual values and horizontal lines the median. ^*^p < 0.05.

To further evaluate the regulatory function of CD39^+^ Treg, we investigated production of the Treg-associated cytokines TGF-β1, IL-10 and IL-17 [13, 34], using the antibody for latency associated peptide (LAP) for detection of TGF-β1 [15]. Unlike the surface expressed effector molecules, the production of TGF-β1 and IL-10 was very similar between the Treg subsets (Figure 4A, 4B). Only in peripheral blood, where the TGF-β1 production was also the highest, the TGF-β1 production was significantly higher in the CD39^+^ Treg subset compared to the CD39^−^ Treg subset (Figure 4A).

Production of IL-17 by a subset of tumor-associated Treg has been suggested to aggravate tumor progression, by the combined effect of tumor-promoting IL-17 secretion and the suppression of IFN-γ secretion originating from Th1 effector T cells [34]. Nevertheless, in our material, IL-17 production did not differ significantly between the Treg subsets (Figure 4C). Furthermore, the IL-17 production by all tumor-associated Treg compared to Treg from unaffected colon tissue was also similar, while IL-10 production by tumor-associated Treg was lower than in Treg from unaffected colon (Supplementary Figure 3).

Hence, in contrast to the data on surface expressed immunoregulatory molecules, the Treg cytokine production does not distinguish the CD39^+^ Treg subset compared to CD39^−^ Treg in the tumor. Instead, immunosuppressive cytokines are secreted to the same extent by both Treg populations.

### CD39^+^ Treg from peripheral blood display high immunosuppressive capacity

**Figure 5:**
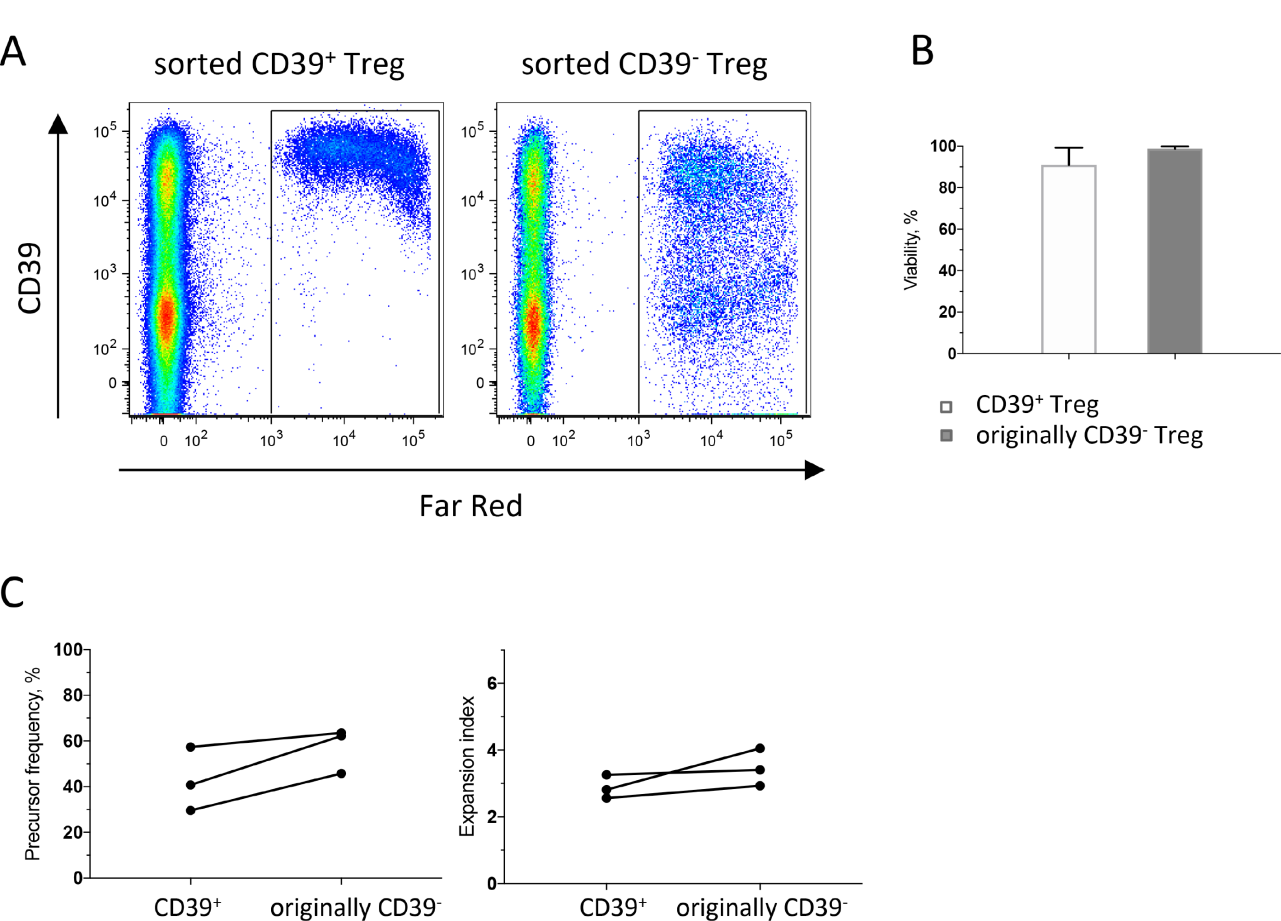
Dynamics of CD39^+^ and CD39^−^ Treg during culture PBMC isolated from buffy coats were enriched for monocytes by immunomagnetic sorting, and for conventional T cells (CD4^+^CD127^+^CD25^−^) and CD39^+^ and CD39^−^ Treg (CD4^+^CD127^lo^CD25^hi^) by flow cytometric cell sorting and sorted Treg subsets were labeled with CellTrace Far Red before culture. Treg: T responders were added at a 1:1 ratio and stimulated with anti-CD3 mAb. Flow cytometry analyses were performed after four days of culture. (**A**) Representative dot-plots gated on CD3^+^ cells, showing cultures with sorted CD39^+^ Treg to the left and sorted originally CD39^−^ Treg to the right. (**B**) Viability of sorted CD39^+^ and originally CD39^−^ Treg after four days of culture. (**C**) Proliferation analysis of sorted CD39^+^ and CD39^−^ Treg, showing precursor frequency to the left and expansion index to the right. Bars represent mean values (SEM) and symbols individual values consistently linked within individuals (*n* = 3).

To expand our findings from the phenotypical experiments and to examine if CD39^+^ Treg might be more immunosuppressive than CD39^−^ Treg, we performed co-culture experiments using peripheral blood from healthy volunteers. By using conventional gating strategies for sorting live Treg combined with CD39 [26], we were able to sort sufficient numbers of CD39^+^ and CD39^−^ Treg with high purity and viability (Supplementary Figure 4). Conventional T cells were cultured with or without CD39^+^ or CD39^−^ autologous Treg and activated by stimulation with anti-CD3. When responder T cell proliferation was assessed in a ^3^H-thymidine incorporation assay after five days of culture, we could demonstrate that CD39^+^ Treg potently suppress the proliferation of autologous responder T cells (Supplementary Figure 5). Also CD39^−^ Treg exhibited suppressive ability, and suppressive ability of both Treg subsets was concentration-dependent. To exclude that the observed difference in suppressive ability between CD39^+^ Treg and CD39^−^ Treg in the ^3^H-thymidine incorporation assay was due to possible differences in viability or proliferation between the subsets, we labeled both Treg subsets with CellTrace Far Red before culture. Somewhat unexpectedly, it was evident from these experiments that a large fraction of the CD39^−^ Treg started to express CD39 during culture (Figure 5A). After four days in culture, 44–59% of the formerly CD39^−^ Treg expressed CD39 (*n* = 3). We conclude that upregulation of CD39 on the formerly CD39^−^ Treg following CD3-mediated activation makes it very hard to compare the suppressive ability of strictly CD39^+^ and CD39^−^ Treg, even after stringent cell sorting.

CD39^+^ and originally CD39^−^ Treg showed similar viability after four days in culture (Figure 5B). The originally CD39^−^ Treg exhibited a slightly higher proliferation compared to CD39^+^, both with regard to precursor frequency, i.e. the fraction of the original population that divided at least once and expansion index, i.e. the fold expansion of all labelled cells during culture (Figure 5C). More interestingly, we also analyzed proliferation of converted CD39^+^ Treg and the Treg that remained CD39^−^, and compared their proliferation with original CD39^+^ Treg (Supplementary Figure 6). These data show that recent CD39^+^ Treg that had converted during culture had the highest proliferation with regard to replication index, i.e. the fold expansion of only the responding cells. Taken together, these data show that CD39 is induced on formerly CD39^−^ Treg upon T cell receptor ligation and may also suggest that CD39^+^ Treg are superior to Treg that remain CD39^−^ in suppressing responder T cell proliferation.

### Cytokine production in cultures with CD39^+^ and CD39^−^ Treg

**Figure 6:**
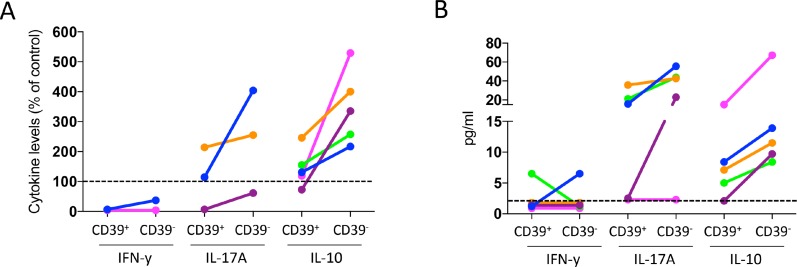
Effect of CD39^+^ and CD39^−^ Treg on cytokine production by conventional CD4^+^ T cells PBMC isolated from buffy coats were enriched for monocytes by immunomagnetic sorting, and for conventional T cells (CD4^+^CD127^+^CD25^−^) and CD39^+^ and CD39- Treg (CD4^+^CD127^lo^CD25^hi^) by flow cytometric cell sorting, and stimulated with anti-CD3 mAb. IFN-β, IL-17A and IL-10 concentration was analyzed by MAGPIX array after two days of culture, with responder cells and sorted CD39^+^ or originally CD39^−^ Treg at a 1:1 ratio (*n* = 5). Data are expressed as % of the cytokine levels compared to cultures without Treg (**A**). Individuals with no detectable cytokine levels in the cultures without Treg were excluded. Cytokine levels in cultures with CD39^+^ and originally CD39- Treg are displayed as absolute values for all individuals (**B**), dotted line indicates lowest detectable concentration. Individuals are consistently color-tagged between graphs.

Production of IFN-γ, IL-13, IL-17A, TNF-α, IL-10, IL-22 and IL-23 was also assessed in co-culture experiments with sorted CD39^+^ and originally CD39^−^ Treg from peripheral blood. TNF-α was secreted to a relatively large extent, ranging between 127 to 539 pg/ml in the cultures with T effector cells alone, but the effects of CD39^+^ and originally CD39^−^ Treg on TNF-α production were variable and inconclusive (data not shown). In the experiments with detectable IFN-γ secretion in cultures with responder cells alone, IFN-γ secretion was suppressed by Treg, and suppression was similar using both Treg subsets (Figure 6A, 6B). In contrast, IL-17A and IL-10 levels were usually higher with Treg in the cultures, compared to responder cells alone. Interestingly, cultures with originally CD39^−^ Treg always contained more IL-10 and IL-17A when compared to cultures with CD39^+^ Treg (Figure 6A, 6B). As cytokine concentrations were low, and not always detectable in the responder cell cultures optimized to measure proliferative responses, we show cytokine levels both in relation to the untreated cultures (Figure 6A) and the absolute values in cultures with CD39^+^ and originally CD39^−^ Treg (Figure 6B). For IL-13, IL-22, and IL-23, only very low or undetectable cytokine concentrations were found (data not shown). We conclude that the Th1-associated cytokine IFN-γ is reduced in culture with both CD39^+^ and originally CD39^−^ Treg, while IL-10 and IL-17A are enriched particularly in cultures with originally CD39^−^ Treg.

### Tumor-infiltrating Treg can be highly immunosuppressive

Due to the limited numbers of Treg that can be isolated from the tumors (Figure 1B) as well as the dynamic expression of CD39 on Treg, it was not possible to compare immunosuppressive ability between intratumoral CD39^+^ and CD39^−^ Treg. Instead, we hypothesized that tumor-infiltrating Treg may have a higher immunosuppressive capacity compared to Treg from unaffected colon and peripheral blood, as an effect of their higher frequencies of CD39^+^ Treg in the tumors. Using the same suppression assay as in the above experiments, we found that the suppression achieved by tumor-associated Treg was variable, and on occasion even inferior compared to Treg from unaffected colon tissue (Supplementary Figure 7). This was independent of the frequencies of CD39−expressing Treg in the tumor versus the unaffected colon tissue *ex vivo*. Treg from peripheral blood of the cancer patients were, however, generally less suppressive compared to Treg isolated from the tissues. Taken together, tumor-infiltrating Treg generally have a potent immunosuppressive potential. Our data also indicate that the immunosuppressive potential of tumor-associated Treg may vary between cancer patients.

## DISCUSSION

In this study, we show that CD39^+^ Treg are enriched in the microenvironment of colon cancer and express phenotypic markers indicating increased turnover and suppressive ability. In addition, CD39^+^ Treg from peripheral blood potently suppress proliferation by conventional T cells. Thus, our results indicate that CD39^+^ Treg may be a superior immunosuppressive population compared to CD39^−^ Treg and suggest that therapies aimed at reducing CD39^+^ Treg activity may be useful to reduce immunosuppression in CRC.

The Treg subsets accumulating in CRC have been characterized as activated effector Treg with suppressive phenotypes [15, 35], but previously these data were hard to reconcile with results from large retrospective studies showing a correlation between higher infiltration of Foxp3^+^ putative Treg and a beneficial patient outcome [16, 17]. These seemingly contradictory observations have now been challenged and new findings indicate two distinct Foxp3^+^ T cell populations in CRC; the suppressive Foxp3^hi^ Treg population, associated with poor prognosis and the non-suppressive Foxp3^lo^ T cell population, associated with good prognosis [18]. In this regard, it is interesting to note that CD39^+^ tumor-infiltrating Treg in our patient cohort express more Foxp3 on a per cell basis than CD39^−^ Treg, suggestive of a more immunosuppressive effector Treg subset associated with poor patient outcome.

The increased frequencies of CD39^+^ Treg in tumors could be due to increased recruitment, proliferation, or retention, or a local conversion from CD39^−^ cells. The higher proliferation rate of CD39^+^ Treg may partly account for their higher frequencies in the tumors, as may chemokine-mediated recruitment. CCR6 and CCR10 are implicated in Treg homing to tumors [29, 30], and were more highly expressed by the CD39^+^ Treg in the tumor. A considerable part of the CD39^+^ Treg in the tumor expressed CCR6 and interestingly for this context, CCR6^+^ regulatory T cells have been associated with superior suppressive activity in oral cancer [30]. Frequencies of CCR10-expressing CD39^+^ Treg were however dramatically lower in the tumor compared to peripheral blood, even though recent findings in human ovarian cancer cell lines show that hypoxia induces CC-chemokine ligand 28 (CCL28) which in turn recruits CCR10-expressing Treg [36]. Since hypoxia also enhances the generation of adenosine [24], co-expression of CD39 and CCR10 by intratumoral Treg would seem likely. However, CCL28 production in CRC tissue is low [37], which may explain the lower migration of CCR10-expressing Treg to the tumors. The expression of adhesion molecules, like integrins and selectins, may also vary between Treg cells, and would also contribute to different homing properties. In addition to increased recruitment of CD39^+^ Treg into the tumor, we cannot rule out increased retention of CD39^+^ Treg. Acquisition of CD39 expression within the tumors may also contribute to the increased frequencies of CD39^+^ Treg. Indeed, CD39 expression can be upregulated in tissues on newly induced or converted Treg subsets [38, 39], and we also show that CD39 can be induced on a subset of CD39^−^ Treg upon stimulation. Taken together, it appears that several factors act in concert to generate the accumulation of CD39^+^ Treg in the tumors.

The phenotypic studies of tumor-infiltrating Treg in relation to CD39 expression were partly based on the immunosuppressive surface molecules analyzed; ICOS, PD-L1 and CTLA-4, which were all increased on CD39^+^ Treg. These are all important Treg effector molecules for immune regulation, and already established or future potential targets for cancer immunotherapy [40, 41]. The ICOS protein is not immunosuppressive in itself, but confers an activation signal to Treg leading to increased suppressive ability [33], while PD-L1 and CTLA-4 confer direct immunomodulation by binding to counter-receptors on T cells and dendritic cells, respectively. In a murine colon carcinoma model, simultaneous blocking of PD-L1 and CTLA-4 was correlated with an enhanced anti-tumor response [42], indicating the significance of higher expression levels of immunosuppressive surface molecules by Treg also in colon cancer patients. Taken together, our data, along with previous studies [13, 30, 34] indicate that the Treg in both tumors and unaffected tissues are a heterogeneous population, yet CD39 expression marks Treg with a phenotype indicating a superior suppressive activity and as such may affect patient outcome. Indeed, the adenosine generated by CD39 enzymatic activity may act in an autocrine fashion and induce increased expression of other suppressive mechanisms [23].

In addition to the net generation of immunosuppressive adenosine, CD39 expression also reduces extracellular ATP levels which in turn reduces the downstream signaling from P2Rs (purinergic ATP receptors) on immune cells and other cell types, e.g. tumor cells. While both adenosine and extracellular ATP are generally higher in the tumor microenvironment compared to normal tissue [43], extracellular ATP is less straightforwardly correlated to tumor growth, compared to adenosine, and can be both tumor promoting and tumor suppressing [43–45]. Consequently the prognostic effect of CD39 expression by Treg in colon cancer is difficult to envision, even though an enriched CD39^+^ Treg population likely shifts the balance to a proportionally higher level of adenosine receptor signaling. Nonetheless, lower total levels of CD39 mRNA in CRC have been associated with improved patient outcome [46]. The total effect of CD39^+^ Treg must also take into account the increased expression of other suppressive surface molecules as discussed above.

Similar to the surface effector molecules on Treg, previous studies strongly indicate the presence of several subsets of Treg based on differential production of cytokines [13, 34]. Interestingly, when analyzed by flow cytometry, the majority of TGF-β1-producing Treg in peripheral blood expressed CD39, but frequencies of TGF-β1-producing Treg were lower in the tissue and more similar in the CD39^+^ and CD39^−^ Treg populations. Also IL-10 was similarly produced by CD39^+^ and CD39^−^ Treg in the tumors. IL-10 is crucial for maintaining the immune homeostasis of the intestinal tract [47] and may thus be constitutively expressed by both Treg subsets. Interestingly though, frequencies of IL-10-producing Treg were lower in the tumor compared to unaffected colon tissue, suggestive of an imbalance in the immune homeostasis in the tumor, as previously indicated in a mouse model of hereditary colon cancer [48]. IL-17 has direct effects on tumor growth [49], increases angiogenesis, and also increases pro-tumorigenic inflammation by acting on mast cells in the gut mucosa [48]. Thus, IL-17-producing Treg may act as a double-edged sword, by both promoting tumor growth and reducing protective Th1-type responses, and this may be particularly relevant in intestinal tumors in close contact with the intestinal flora [34, 50]. In our material though, there was no clear correlation between IL-17 production and CD39 expression in Treg from colon tumors. Treg in CRC have been shown to use PD-L1, IL-10 and TGF-β for suppression of conventional T cells [14, 15] and our current study also identify these effector mechanisms in CD39^+^ Treg. Of these, only PD-L1 expression was enriched in the CD39^+^ population, and we suggest that CD39^+^ Treg in CRC preferentially use more PD-L1 and CTLA-4 compared to CD39^−^ Treg, together with their contribution towards generation of adenosine, to suppress conventional T cell functions.

The flow cytometry analyses showing similar IL-10 and IL-17 production in CD39^+^ and CD39^−^ Treg are not easily reconciled with our co-culture experiments showing more IL-10 and IL-17 in cultures with originally CD39^−^ sorted Treg. Generally, the overall IL-17A levels in the *in vitro* co-culture suppression assays were usually higher when Treg were present in the cultures. This may result from a suppressed IFN-γ secretion by Treg and a subsequent unleashed IL-17A secretion when the Th1 signal is removed. Furthermore, the higher IL-17A secretion levels detected in the co-cultures with originally CD39^−^ Treg may suggest that CD39^−^ Treg are inferior at suppressing IL-17A secretion compared to CD39^+^ Treg. Alternatively, the originally CD39^−^ Treg may actually have the ability to induce IL-10 and IL-17A in responder T cells.

In our *in vitro* co-culture suppression assays performed on peripheral blood, we observed higher proliferation among converted CD39^+^ Treg compared to the originally CD39^−^ Treg that remained CD39^−^, and a lower total proliferation in cultures with CD39^+^ Treg. Similar findings have been reported in Treg isolated from synovial fluid from patients with rheumatoid arthritis [22], but using less stringent criteria to identify Treg. However, the conversion of originally CD39^−^ Treg to CD39^+^ upon activation, makes it hard to analyze the suppressive activity of CD39^−^ Treg. We also show that tumor-infiltrating Treg can be highly suppressive *in vitro*. Since cultures with CD39^+^ Treg contained lower levels of IL-17A compared to cultures with originally CD39^−^ Treg, the high frequencies of *ex vivo* CD39^+^ Treg in the tumors may influence the Th_1_:Th_17_ ratio and thereby affect patient outcome [8]. We argue that the differences in suppressive ability of tumor-infiltrating Treg on responder T cell proliferation are probably due to individual variation between patients, due to e.g. tumor heterogeneity and environmental factors.

The addition of immunotherapy to the treatment options for colon cancer would potentially have a great impact on patient outcome and survival. Earlier attempts at releasing the suppression of conventional CD4^+^ and CD8^+^ T cells in CRC with anti-PD-1 and anti-CTLA-4 blockade, which have shown excellent results in therapy of advanced melanoma and prostate cancer, have failed, and so far no specific immunotherapy has been registered for CRC [51]. However, recent studies indicate that anti-PD-1 therapy may be an option in microsatellite instability high (MSI-H) tumors [51, 52]. Further broadening of the immunotherapy arsenal is thus warranted, and the variations recorded in intratumoral CD39^+^ Treg frequencies in some patients in our cohort set the ground for future retrospective follow-up studies on the importance of intratumoral CD39^+^ Treg for patient survival. CD39 activity has not yet been implicated in Treg immunosuppressive function in colorectal cancer, as it has in breast cancer where the addition of both an A2R-antagonist and an anti-CD39 antibody inhibited the suppressive effect of Treg on IFN-γ production by Th1 and CD8^+^ T cells *in vitro* [39]. In addition, a neutralizing human anti-CD39 antibody has been shown to inhibit tumor cell-mediated immunosuppression in pre-clinical evaluations [53]. An attempt at targeting CD39 on Treg may be particularly beneficial due to the high expression of CD39 on tumor-infiltrating Treg. Targeting of CD39 on Treg may also be beneficial due to the low CD39 expression on CD4^+^CD25^−^ and CD4^+^CD25^int^ tumor-infiltrating effector T cell subsets as previously shown [9]. In conclusion, our results suggest that CD39^+^ Treg in colon adenocarcinomas are a potent immunosuppressive population, and indicate that immunotherapies aimed at targeting CD39^+^ Treg may be promising against colon cancer.

## MATERIALS AND METHODS

### Volunteers

The study was performed with the permission of the Regional Research Ethics Committee of West Sweden, and informed consent was obtained from all patients. Peripheral blood, unaffected colon tissue, and tumor were collected from 46 consecutive colon adenocarcinoma patients (25 males and 21 females, aged 46–93) undergoing curative colectomy. None of the patients had undergone radiotherapy or chemotherapy for at least 3 years prior to colectomy, and none suffered from autoimmune disease. Patient details and tumor characteristics are presented in Supplementary Table 1.

### Isolation and stimulation of lymphocytes

Blood from patients was collected in heparinized tubes, and peripheral blood mononuclear cells (PBMC) were isolated by Ficoll-Paque (Pharmacia) density-gradient centrifugation. A piece of the tumor and unaffected colon mucosa was collected at the time of surgery. The tissue was immediately placed in ice-cold PBS and used for isolation of lamina propria lymphocytes (LPL) by enzymatic digestion after removal of epithelial cells, essentially as described [54]. Due to enzymatic cleavage of CD25 by collagenase, however, we used 36 μg/ml of Liberase (Roche) for 1 hour. For cytokine detection, isolated cells were incubated with 10 ng/ml of PMA and 0,6 μg/ml Ionomycin directly after isolation. Golgi-stop was added simultaneously, and stimulated lymphocytes were harvested 9–11 hours later. For phenotypic flow cytometry analyses and cell sorting experiments, isolated cells were kept overnight at 4° C and then stained with selected antibody-fluorochrome conjugates.

### Flow cytometry analyses

Flow cytometry analyses were performed on single cells after excluding dead cells with Live/Dead fixable aqua dead cell stain kit (Molecular Probes). Data acquisition was performed on a LSRII flow cytometer (BD Biosciences), equipped with FACS Diva software (BD Biosciences) and analyzed using FlowJo software (TreeStar Inc). The following antibodies and reagents were used: CD39−FITC (clone A1) from AbD Serotec; CD3-AF700 (clone UCHT1), CD4-PerCP (clone OKT4), LAP(TGF-β1)-BV421 (clone TW4-2F8), IL-10-PE (clone JES3-9D7) and CCR8-PE (clone L263G8) from BioLegend; CD25−BV650 (clone M-A251), CD8-BV711 (clone RPA-T8), CD127-PE (clone HIL-7R-M21), CD45RA−PE (clone HI100), Ki67-AF700 (clone B56), CD278-BV711 (clone DX29), CD274-BV786 (clone MIH1), CD-69-PE (clone FN50), CTLA-4-PE (clone BN13), CXCR3-AF700 (clone 1C6/CXCR3), CCR4-V450 (clone 1G1), CCR10-BV421 (clone 1B5) and CCR5-BV711 (clone 2D7/CCR5) from BD Biosciences; Foxp3-APC (clone PCH101), IL-17-PE (clone eBio64DEC17) and CCR6-PE (clone R6H1) from eBioscience; and CCR9-PE (clone 248621) from R&D Systems. To stain for Foxp3, Ki67, CTLA-4, TGF-β1, IL-10 and IL-17, we used the Foxp3 Staining Buffer Set (eBioscience) for intracellular staining. Isotype controls were used as reference to determine marker expression.

### Flow cytometry cell sorting

Flow cytometry cell sorting was used for the isolation of CD4^+^CD127^lo^CD25^hi^ Treg from PBMCs, unaffected colon tissue and tumor and CD4^+^CD127^+^CD25^−^ conventional T cells from PBMCs and unaffected colon tissue [26]. Buffy coats from healthy volunteers were pre-enriched for CD4^+^ T cells via negative selection using immunomagnetic sorting with EasySep Human CD4^+^ T cell Isolation (Stemcell). Enriched CD4^+^ T cells were sorted using the following antibodies and reagents: Live/Dead fixable aqua dead cell stain kit from Molecular Probes; CD4-PerCP (clone OKT4) from BioLegend; CD8-BV711 (clone RPA-T8), CD25−BV650 (clone M-A251) and CD127-PE (clone HIL-7R-M21) from BD Biosciences; CD127-FITC (clone eBioRDR5) from eBioscience; and CD39−FITC (clone A1) from AbD Serotec, on a FACS Aria (BD Biosciences) equipped with FACS Diva software (BD Biosciences). FMO and isotype controls were used as reference to determine marker expression. The sorted cells were manually counted with Trypan Blue solution and mean viability of sorted cells was 94% for Treg and 92% for conventional T cells.

### Suppression assays

Suppressions assays were conducted essentially as described [55]. Monocytes were enriched from autologous PBMC using immunomagnetic sorting with CD14 MicroBeads (Miltenyi Biotec) according to the manufacturer’s description, and used as accessory cells. The viability of sorted monocytes was always >95%, and the purity >97%. 2 × 10^4^ isolated monocytes were pre-incubated for either 5 or 24 hours depending on experimental setup (5 hours for experiments with sorted PBMCs from healthy individuals and 24 hours for experiments with sorted tissue cells from colon cancer patients) in round-bottomed 96-well plates in complete medium (Iscove’s medium supplemented with 5% AB^+^ serum, 2 mM L-glutamine and 50 μg/ml gentamicin (Life Technologies). 10^4^ responder T cells (conventional CD4^+^CD127^+^CD25^−^ T cells) and various numbers of Treg/Treg subsets were added to the wells, and stimulated with 1 μg/ml of soluble anti-CD3mAb (clone OKT-3 from eBioscience) in a total volume of 200 μl complete medium. Treg/Treg subsets were added to create Treg:T responder ratios of 1:1, 1:2, 1:4, and 1:8. Cell culture supernatant (50 μl) was removed from each well after 48 h and replaced with fresh medium. Replicate supernatants were pooled and stored at −70° C. After an additional 72 hours of culture, T cell proliferation was measured by pulsing the cells with 1 μCi [^3^H] thymidine per well (Amersham Biosciences) for 6 h. Incorporated radioactivity was analyzed in a scintillation counter (Perkin Elmer).

In some suppression experiments, sorted CD39^+^ and CD39^−^ Treg were labeled with CellTrace Far Red (Life Technologies) according to the manufacturer’s description before culture. Treg subsets and responder cells were then added at a 1:1 ratio. Cells were harvested and analyzed by flow cytometry after four days of culture.

### Cytokine analysis

Cytokine concentrations in culture medium were analyzed using the Luminex bead-based Multiplex immunoassay with reagents from Bio-Rad. Panels were customized into one 3-plex (IFN-γ, IL-17A and IL-13 within the human Group I panel from Bio-Rad) and one 4-plex (IL-10, TNF-α, IL-22 and IL-23 within the human Th17 panel from Bio-Rad). Plates were analyzed on the MAGPIX Multiplexing System (Luminex Corporation).

### Statistical methods

Wilcoxon matched-pairs signed rank test was used to compare data obtained from the same individual. *P* values of < 0.05 were considered significant.

## SUPPLEMENTARY MATERIALS FIGURES AND TABLE



## Abbreviations

CCL28: CC-chemokine ligand 28
CRC: colorectal cancer
CTLA-4: cytotoxic T-lymphocyte-associated protein 4
ICOS: inducible costimulatory molecule
LAP: latency associated peptide
MFI: mean fluorescence intensity
PBMC: peripheral blood mononuclear cells
PD-1: programmed death-1
PD-L1: programmed death-ligand 1
Th1: T helper 1
Th17: T helper 17
Treg: regulatory T cells
