# Supplementary material for: CD39+ regulatory T cells accumulate in colon adenocarcinomas and display markers of increased suppressive function
Source: Oncotarget. 2018 Dec 11;9(97):36993–7007. doi: 10.18632/oncotarget.26435 (PMC6319332; doi:10.18632/oncotarget.26435)
Supplement: Supplementary file 1 [file oncotarget-09-36993-s001.pdf]

CD39$^+$ regulatory T cells accumulate in colon adenocarcinomas and display markers of increased suppressive function

SUPPLEMENTARY MATERIALS

Supplementary Table 1: Characteristics of the colon cancer patients included in the study

		females	males
	n	21	25
	age	62–88	46–93
Tumor location	cecum	6	7
	ascending colon	10	6
	transverse colon	2	1
	descending colon	1	3
	sigmoideum	2	8
Differentiation grade	high	1	9
	medium	8	10
	low	11	5
	mucinous	1	1
Tumor stage	T1	-	-
	T2	2	4
	T3	14	17
	T4	5	4
Lymph node spread		12	14
Distant metastases		-	3

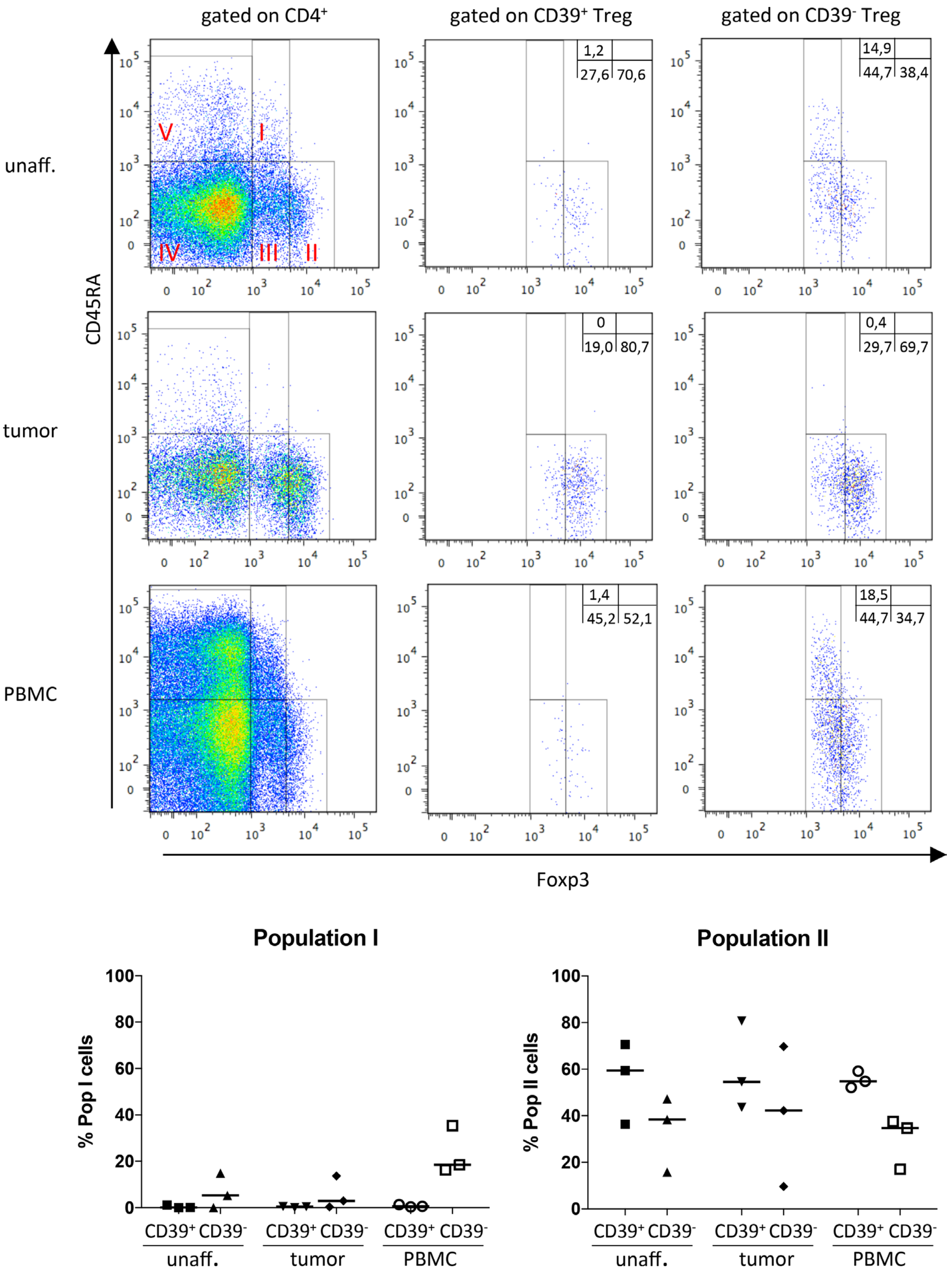

Supplementary Figure 1: Expression of CD45RA and Foxp3 by CD39⁺ and CD39⁻ Treg. Single-cell suspensions were isolated from unaffected colon lamina propria, tumor, and PBMCs. CD39⁺ and CD39⁻ Treg were analyzed for expression of CD45RA and Foxp3 using flow cytometry, and classified into three subpopulations; CD45RA⁺Foxp3^{lo} (pop. I), CD45RA⁻Foxp3^{hi} (pop. II), and CD45RA⁻Foxp3^{lo} (pop. III). Representative FACS-plots show data from unaffected colon lamina propria, tumor and PBMCs, and a compilation of data below for population I and II. Representative FACS-plots gated on CD4⁺ T cells showing the original classification into five different subpopulations (pop. I-–V) [27]. Symbols represent individual values and horizontal lines the median (n = 3).

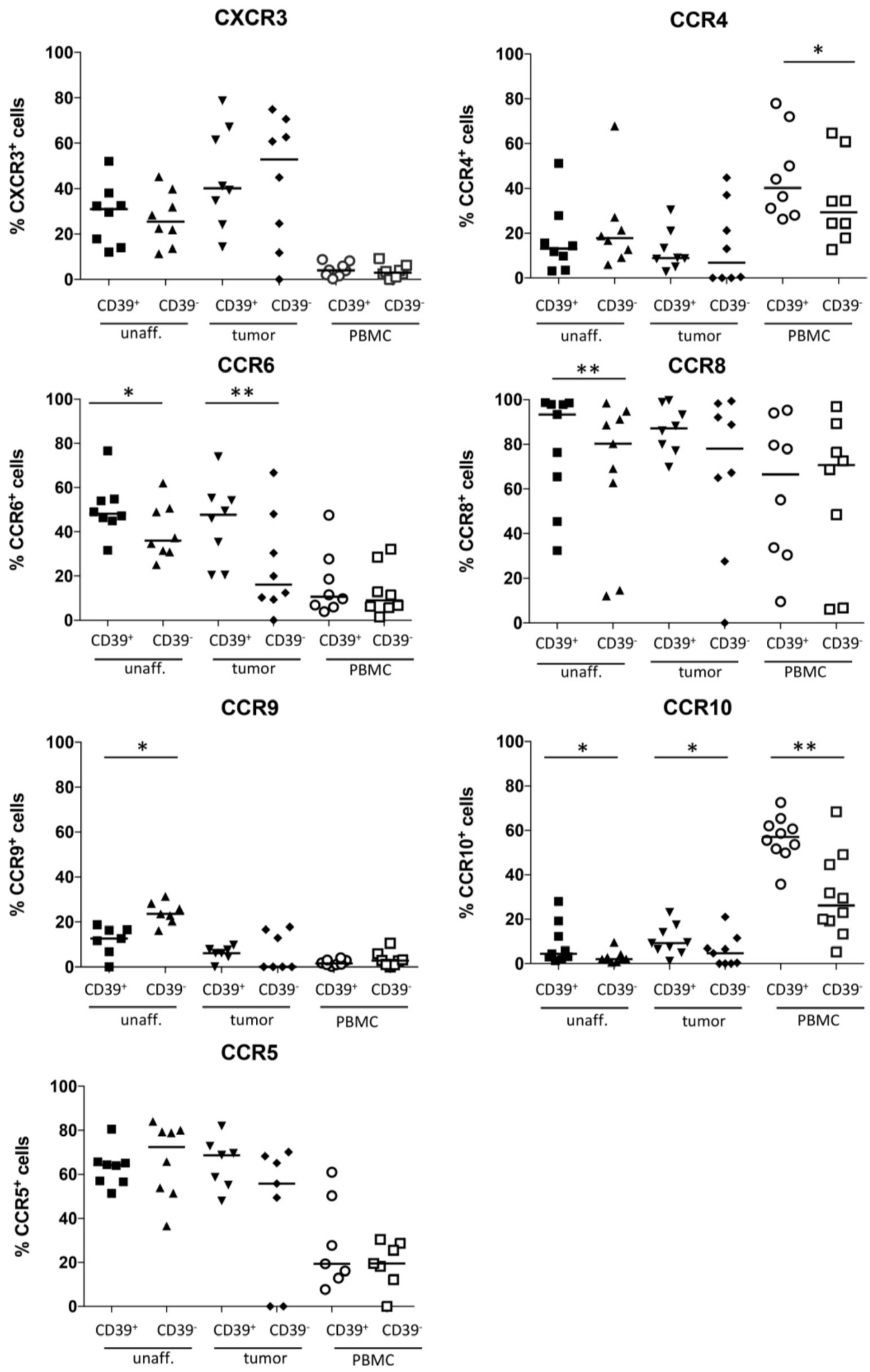

Supplementary Figure 2: Expression of chemokine receptors by CD39⁺ and CD39⁻ Treg. Single-cell suspensions were isolated from unaffected colon lamina propria, tumor, and PBMCs. CD39⁺ and CD39⁻ Treg were analyzed for expression of chemokine receptors by flow cytometry using isotype controls to determine chemokine receptor-specific staining. A compilation of data for the various chemokine receptors is shown. Symbols represent individual values and horizontal lines the median (n = 8–10). $^{*}p < 0.05$, $^{**}p < 0.01$.

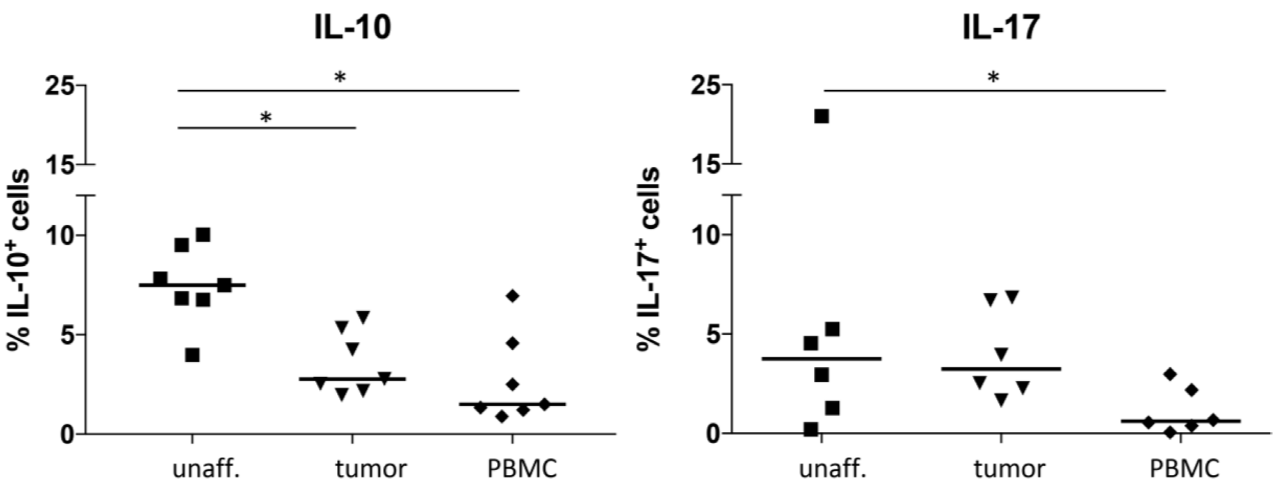

Supplementary Figure 3: IL-10- and IL-17-production by Treg. Single-cell suspensions were isolated from unaffected colon lamina propria, tumor, and PBMCs, and stimulated with PMA/Ionomycin. The frequencies of IL-10- and IL-17-producing cells among $CD4^{+}CD25^{hi}Foxp3^{+}$ Treg were analyzed by flow cytometry using unstimulated cells as reference to determine cytokine-specific staining. Symbols represent individual values and horizontal lines the median (n = 6–7). $^{*}p < 0.05$.

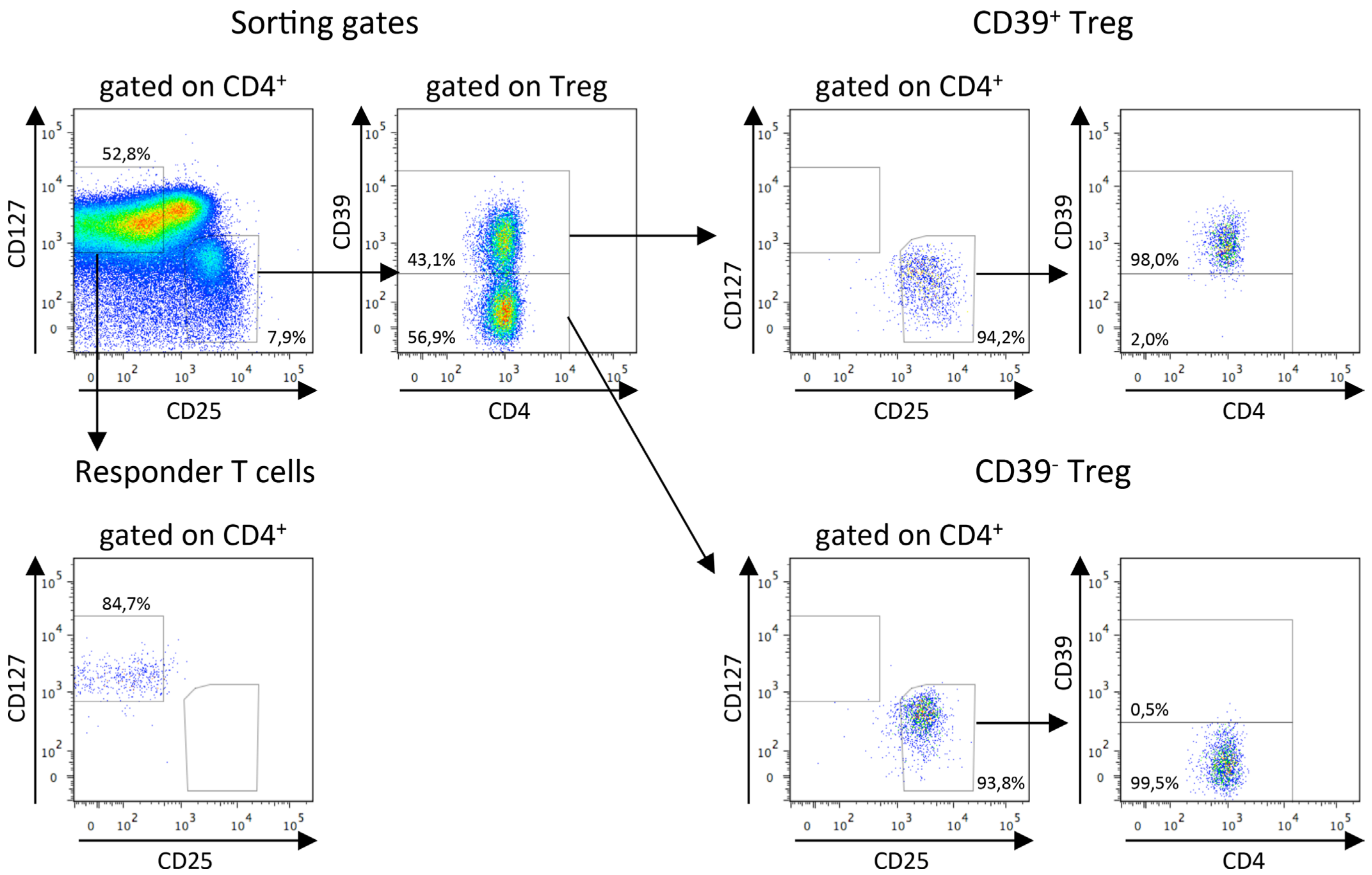

Supplementary Figure 4: Gating strategy for sorting of conventional CD4+ T cells, and $CD39^{+}$ and $CD39^{-}$ Treg. Single-cell suspensions were isolated from buffy coats, stained, and sorted using flow cytometry. Representative FACS-plots show sorting strategies and purity gates for sorted populations.

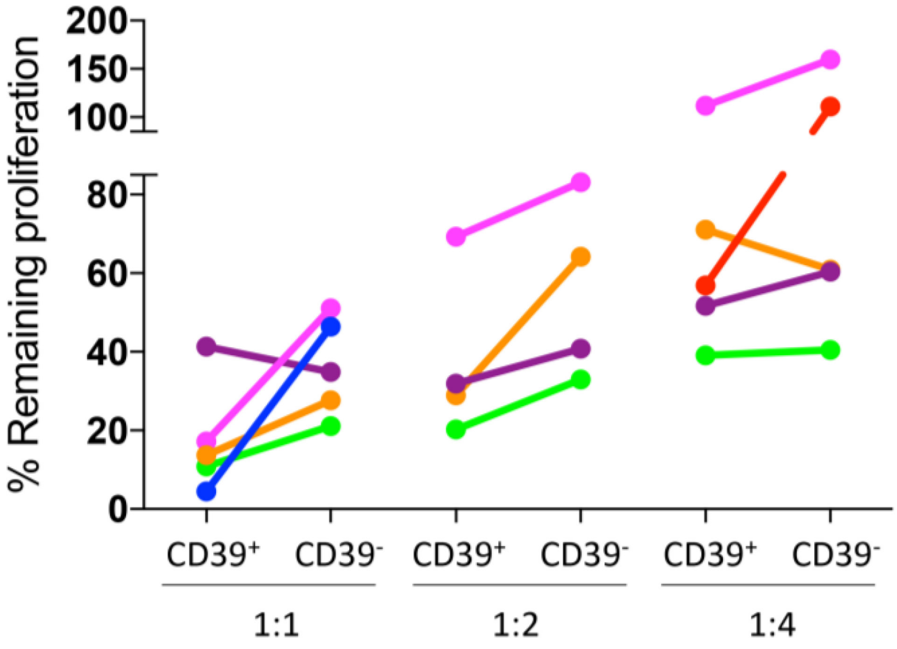

Supplementary Figure 5: Effect of CD39$^+$ and CD39– Treg on conventional CD4$^+$ T cell proliferation. PBMC isolated from buffy coats were enriched for monocytes by immunomagnetic sorting, and for conventional T cells (CD4$^+$CD127$^+$CD25$^-$) and CD39$^+$ and CD39$^-$ Treg (CD4$^+$CD127^{lo}CD25^{hi}) by flow cytometric cell sorting, and stimulated with anti-CD3 mAb. Suppression of responder CD4$^+$ T cell proliferation by sorted autologous CD39$^+$ and originally CD39$^-$ Treg in various ratios was measured by 3H-thymidine incorporation day 5. Data are expressed as remaining proliferation compared to proliferation values in cultures without Treg and different colors are used to indicate individual donors (n = 6).

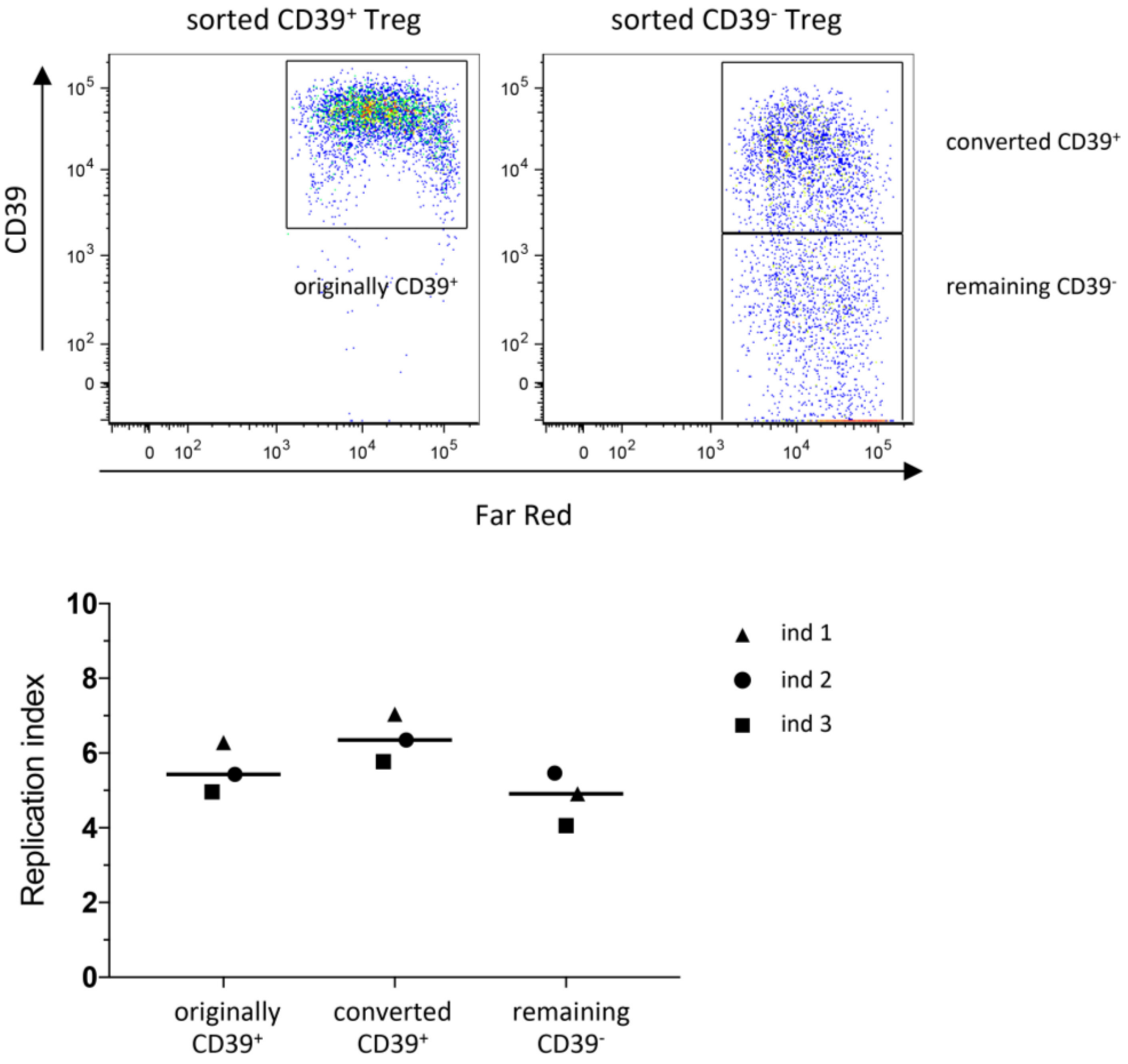

Supplementary Figure 6: Proliferation characteristics of CD39$^+$ and CD39$^–$ Treg during culture. PBMC isolated from buffy coats were enriched for monocytes by immunomagnetic sorting, and for conventional T cells (CD4$^+$CD127$^+$CD25$^–$) and CD39$^+$ and CD39$^–$ Treg (CD4$^+$CD127l^{o}CD25^{hi}) by flow cytometric cell sorting and sorted Treg subsets were labeled with CellTrace Far Red before culture. Treg: T responders were added at a 1:1 ratio and stimulated with anti-CD3 mAb. Flow cytometry analyses were performed after four days of culture. Upper panel shows representative dot-plots gated on CD3$^+$Far Red$^+$ cells of cultures with sorted CD39$^+$ Treg to the left and sorted originally CD39$^–$ Treg to the right, the latter constituting both converted CD39$^+$ Treg and Treg that remained CD39$^–$. Lower panel shows replication index of corresponding Treg subsets. Symbols represent individual values and different symbols are used to indicate individual donors (n = 3).

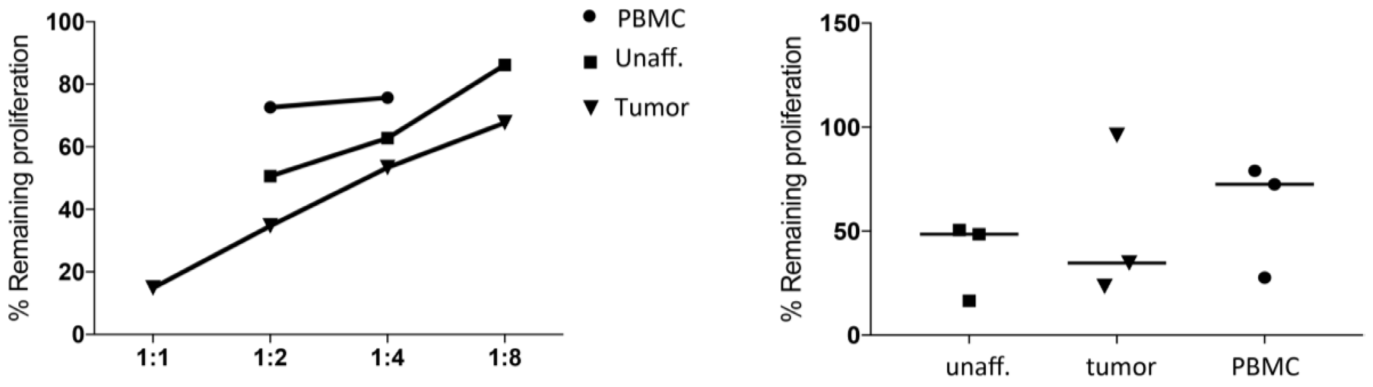

Supplementary Figure 7: Effect of tumor-infiltrating Treg on conventional CD4⁺ T cell proliferation. Single-cell suspensions were isolated from unaffected colon lamina propria, tumor and PBMCs, and PBMCs were enriched for monocytes by immunomagnetic sorting. Isolated cells from unaffected colon lamina propria were enriched for conventional T cells ($CD4^+CD127^+CD25^-$) and Treg ($CD4^+CD127^{lo}CD25^{hi}$), and isolated cells from tumor and peripheral blood were enriched for Treg, all by flow cytometric cell sorting, and later stimulated with anti-CD3 mAb. Suppression of responder $CD4^+$ T cell proliferation by autologous Treg in various ratios was measured by ^{3}H-thymidine incorporation day 5. Data are expressed as remaining proliferation compared to proliferation values in cultures without Treg. Data from one selected patient to the left and a compilation of data to the right, displaying one observation per patient, at the lowest Treg:T responder ratio. Symbols represent individual values and horizontal lines the median (*n* = 3).
